# Using CERES-Maize and ENSO as Decision Support Tools to Evaluate Climate-Sensitive Farm Management Practices for Maize Production in the Northern Regions of Ghana

**DOI:** 10.3389/fpls.2017.00031

**Published:** 2017-01-26

**Authors:** Dilys S. MacCarthy, Samuel G. K. Adiku, Bright S. Freduah, Francis Gbefo, Alpha Y. Kamara

**Affiliations:** ^1^Soil and Irrigation Research Centre, University of GhanaKpong, Ghana; ^2^Department of Soil Science, University of GhanaLegon, Ghana; ^3^Department of Crop Science, University of GhanaLegon, Ghana; ^4^R4D Unit, International Institute of Tropical AgricultureIbadan, Nigeria

**Keywords:** DSSAT model, ENSO, maize production, nitrogen productivity, Northern Ghana, water productivity

## Abstract

Maize (*Zea mays*) has traditionally been a major cereal staple in southern Ghana. Through breeding and other crop improvement efforts, the zone of cultivation of maize has now extended to the northern regions of Ghana which, hitherto, were the home to sorghum and millet as the major cereals. Maize yield in the northern Ghana is hampered by three major biophysical constraints, namely, poor soil fertility, low soil water storage capacity and climate variability. In this study we used the DSSAT crop model to assess integrated water and soil management strategies that combined the pre-season El-Niño-Southern Oscillation (ENSO)-based weather forecasting in selecting optimal planting time, at four locations in the northern regions of Ghana. It could be shown that the optimum planting date for a given year was predictable based on February-to-April (FMA) Sea Surface Temperature (SST) anomaly for the locations with *R*^2^ ranging from 0.52 to 0.71. For three out of four locations, the ENSO-predicted optimum planting dates resulted in significantly higher maize yields than the conventional farmer selected planting dates. In Wa for instance, early optimum planting dates were associated with La Nina and El Niño (Julian Days 130-150; early May to late May) whereas late planting (mid June to early July) was associated with the Neutral ENSO phase. It was also observed that the addition of manure and fertilizer improved soil water and nitrogen use efficiency, respectively, and minimized yield variability, especially when combined with weather forecast. The use of ENSO-based targeted planting date choice together with modest fertilizer and manure application has the potential to improve maize yields and also ensure sustainable maize production in parts of northern Ghana.

## Introduction

The northern regions of Ghana, and indeed the Sahel zones of West Africa have traditionally been home to the small grain cereals such as millet and sorghum. This, apparently is due to their hardiness and ability to withstand low soil fertility and poor water holding capacity (Singh and Singh, 1995) of the dominantly Low Activity Clay (LAC) soils. Over the past few decades, however, maize (*Zea mays*), a major staple crop largely cultivated in southern Ghana has now been extended to the northern regions, due to breeding efforts that target yield response to small amounts of fertilizer among others. Despite these efforts, maize yields continue to be low, ranging from 800 to 1800 kg ha^−1^ without and with fertilizer application (Ragasa et al., 2014). With increased nitrogen application of 90 kg N ha^−1^ or more under good rainfall, maize yields in northern Ghana reached 4500 kg ha^−1^ (Naab et al., 2015). Low soil fertility, is therefore one of the major constraints.

Increasing variability in climate (Laux et al., 2009) has also confounded the problem of low fertility, making investments into inputs and innovative technologies less attractive to smallholders (Hansen, 2005). Increased rainfall variability, especially at the onset of the season has led to staggered planting by farmers to spread the risk of total failure from a single planting date choice. Staggering planting dates is also a way farmers who lack farm labor use family labor more efficiently. The farmers' reliance on their indigenous knowledge for planning farming operations to address the climate change and variability challenge has increasingly been ineffective, as some of the indicators are biological and hence not permanent (Roncoli et al., 2001). Additionally, low water productivity associated with the soils is due to the predominantly coarse-textured shallow soils that render crops vulnerable to agricultural drought (the inability of soil water storage to meet crop water requirements) during some periods in the growing season, even if seasonal rainfall amounts were adequate. The problems enumerated here are wide spread in countries located in Guinea and Sudan savannah and sahelian zones of Sub-Sahara Africa and many other parts of the world with similar climate. In this study, Northern Ghana was used as an example, hence, the output will be applicable to countries with similar climatic and soil characteristics.

Most of the efforts to increase maize yields have been directed to the alleviation of the fertility constraint by promoting fertilizer use (Kombiok and Clottey, 2003; Fosu et al., 2004; Naab et al., 2015). This approach alone, has, however, not resolved poor yields in smallholder systems. With regard to climate variability impact, it is worth noting that studies have shown that some fore-knowledge of the coming season's potential as well as onset could be an important guide to successful farming (Sivakumar, 1988). Proactive planning is required especially with regard to planting date choice to optimize yields for the given season.

Elsewhere, the value of seasonal rainfall forecast to facilitate agricultural decision making has been demonstrated (Carberry et al., 2000; Hansen and Jones, 2000; Jagtap et al., 2002). They concluded that all El Niño events are not equal in their regional manifestation. In East Africa, Amissah-Arthur et al. (2002) established a relationship between El-Niño-Southern Oscillation phenomenon (ENSO) mean seasonal rainfall. Indeje et al. (2000) in their work also indicated that ENSO plays a significant role in determining monthly and seasonal rainfall patterns in East Africa. Climate forecast studies are somewhat fewer in West Africa but mention could be made of Ingram et al. (2002). In Ghana, as in many countries in West Africa, increasing number of studies are now conducted on the usefulness of seasonal forecasting for guiding farm decisions. Starting from 1994, Opoku-Ankomah and Cordrey (1994) linked seasonal rainfall in several parts of Ghana to the SST anomaly of the Atlantic. This was followed by works by Adiku and Stone (1995), Adiku et al. (1997), Adiku et al. (2007), McSweeney et al. (2010) and Mawunya et al. (2011) which all linked seasonal rainfall in Ghana to ENSO. Indeed, the seasonal rainfall in Ghana varies considerably on inter annual and inter decadal time scale. The most well documented cause of these variations is the El Nino southern Oscillation (ENSO) (McSweeney et al., 2010). Their findings showed that the ENSO correlated well with rainfall in southern Ghana, with the cold or negative phase (La Nina) leading to above normal seasonal rainfall, and the warm or positive phase (El Niño) leading to below normal seasonal rainfall. The need to explore weather forecast to support farming operations such as planting date choice in the face of climate change and variability cannot be overemphasized (Laux et al., 2009). Early but erratic onset of the rains may deceitfully lure some farmers to begin planting, only to experience early season dry spell and poor emergence, establishment and crop failure. Long delays in planting may be equally adversarial, especially, if the seasons tends to be short.

**Figure 1:**
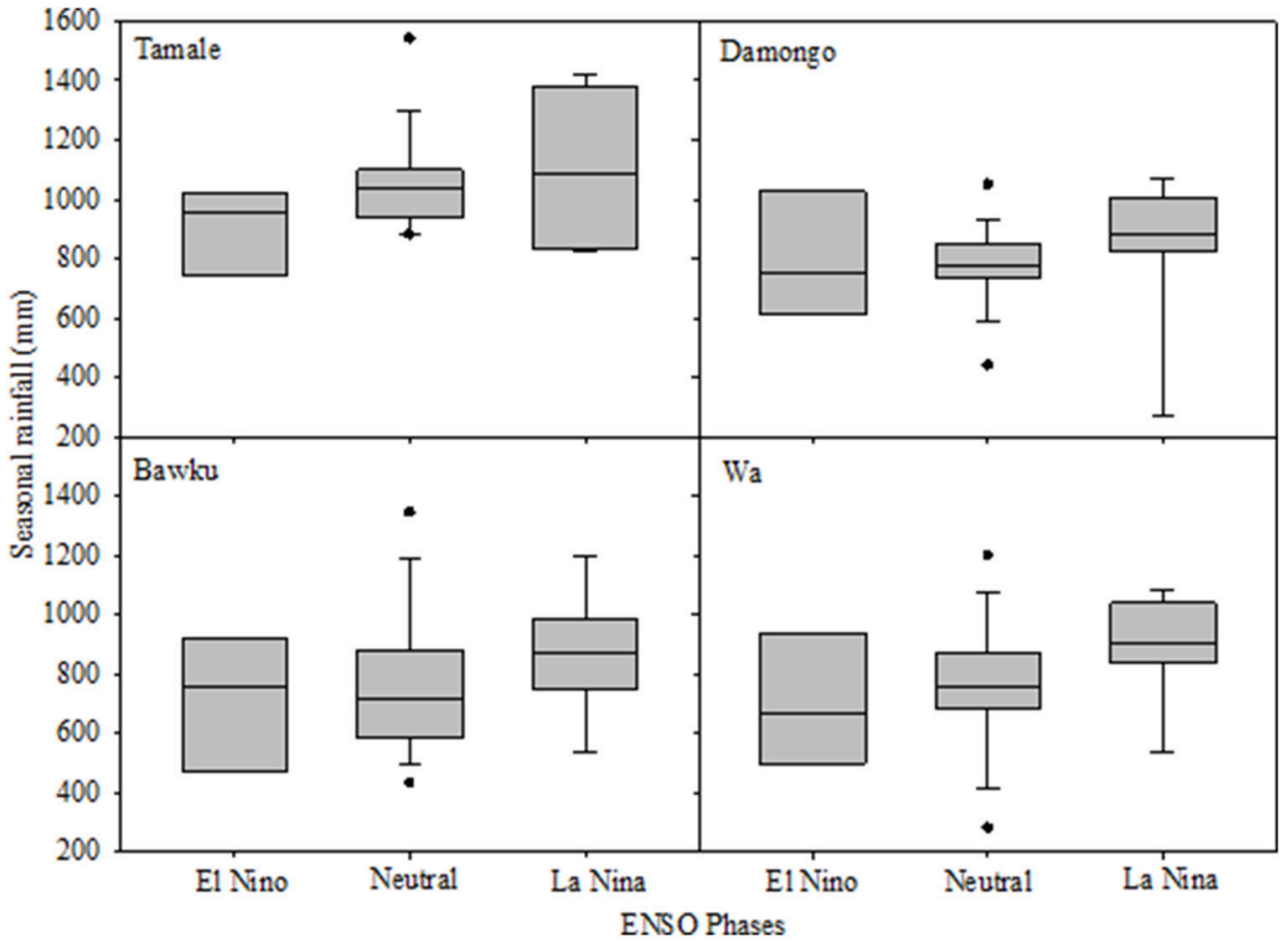
**Box plots of seasonal rainfall distributions according to the ENSO phases (1980–2009) for 4 sites in northern Ghana**. Each box in the graph shows the distribution of rainfall for a specific ENSO phase. The boundary of the box closest to zero indicates the 25th percentile, a line within the box marks the median, and the upper boundary of the box indicates the 75th percentile. Whiskers above and below the box indicate the 95th and 5th percentiles.

Though ENSO-based weather forecast skills are not always high (Shin et al., 2009), the plot of seasonal rainfall vs. pre-season ENSO for the 4 locations of interest to this study shows promise for further investigation (Figure 1).

With regard to within-season agricultural drought, alternative soil and water management practices must be sought to reduce the adverse effects, since irrigation is not an option for these predominantly resource poor farmers. It is known, that soil management practices such as residue retention or mulching can minimize soil evaporation as well as reduce runoff and increase infiltration, leading to increased soil water storage. This should reduce agricultural drought in the face of rainfall variability. Further, it has been shown that increased soil organic matter through amending soils with organic resources can increase the drought resistance of agricultural soils (Hudson, 1994). Farmers in the northern regions of Ghana do not often retain residues on their fields, but instead burn them off before season onset.

It is, therefore, hypothesized that an integrated management system that combines (i) fore-knowledge of the season's potential to minimize climate variability effect on yield, (ii) improved soil management to enhance water use efficiency, and (iii) fertilizer application to address the fertility constraint will form the basis for a sustained maize production in the northern regions of Ghana. The combination of these strategies for successful maize production in the northern regions of Ghana, and many other tropical countries has hitherto not been assessed. Though the study focuses on Northern Ghana, the findings will be relevant to many tropical countries with similar climatic and soil characteristics. The effective evaluation of such options under multi-location and multi-year situations would require a modeling tool such as DSSAT that can integrate crop genotype, soil profile data, weather data and crop management information in determining crop yields. In Ghana, CERES-maize model in DSSAT has been calibrated and used to assess the effects of agronomic practices such as fertilizer and manure application on maize yields in some locations including northern Ghana (Dzotsi et al., 2010; Fosu et al., 2012; MacCarthy et al., 2012).

The aim of this paper is two-fold: First, we explored the extent to which the pre-season ENSO can be used as a basis for selecting the optimum maize planting date for maize production in four (4) major farming zones in the northern regions of Ghana (Tamale, Damongo, Wa, and Bawku). Second, we assessed the combined effects of various seasonal forecast and soil fertility management strategies on the long-term maize productivity in these farming zones using the CERES-maize model.

## Materials and methods

### Locations and physiography

**Figure 2:**
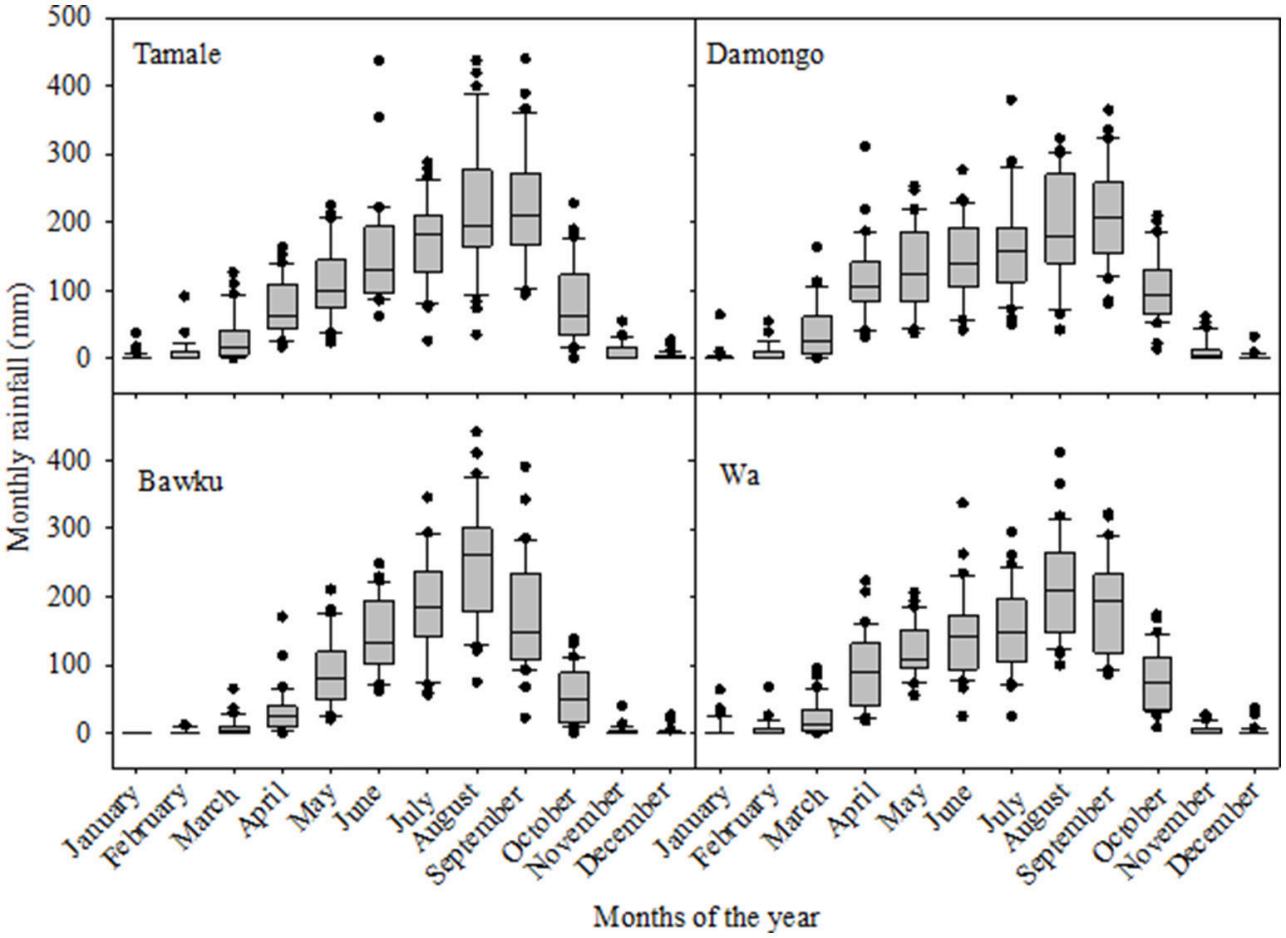
**Annual monthly rainfall distribution for the 4 study sites in northern regions of Ghana**. Each box in the graph shows the distribution of rainfall over the 30 years simulation period. The boundary of the box closest to zero indicates the 25th percentile, a line within the box marks the median, and the upper boundary of the box indicates the 75th percentile. Whiskers above and below the box indicate the 95th and 5th percentiles.

The four study sites are all located in the northern regions of Ghana with Tamale and Damongo in the Northern Region, Bawku in the Upper East and Wa in the Upper West Region. Tamale, Damongo, and Wa lie in the Guinea Savanna agro-ecology while Bawku is characterized by Sudan savanna agro-ecology. The sites were selected to represent a range of climate and soil variability conditions commonly observed in those regions. The rainfall gradient ranges from 900 mm (Bawku) to 1400 mm (Damongo) and the distribution is largely mono-modal at all locations. The growing season in the Guinea Savanna zone is between 5 and 6 months (May to October), followed by dry conditions till the next raining season in the Guinea Savanna zone whereas in the Sudan Savanna, rainfall begins in May and spans between 4 and 5 months. In the Guinea Savanna ecology, annual average temperature is 32°C with the vegetation mainly consisting of grassland with scattered trees while the Sudan Savanna is characterized by a continuum of grassland with scattered shrubs with average annual temperature of 33°C. Monthly rainfall distribution at the 4 sites over the period 1980 to 2009 (30 years) is shown in Figure 2. The soils, which are mainly coarse-textured, have depths varying from very shallow (less than 40 cm) at Bawku to 75 cm at Damongo. The soils are generally poor in fertility with soil organic carbon content <0.5%, and with iron concretions present at depth of about 30 cm.

The farming system is predominantly based on smallholder cereal-legume cultivation in both agro-ecologies. Traditionally, crop planting is preceded by bush burning but increasingly, conventional tillage is gaining popularity. Maize management practices are usually sub-optimal. Even though Ministry of Food and Agriculture's (MoFA) N recommendation for these regions is 60 kg N ha^−1^, most farmers apply less than 30 kg ha^−1^ on the average and the average planting density used on these smallholders' fields is 3.5 plants m^−2^ as against 6.25 plants m^−2^ recommended by MoFA.

### Maize model description

The CERES–maize module, which is a component of the Decision Support System for Agro- technological Transfer (DSSAT), was used in this study to simulate maize yield under variable weather, soil conditions and management for the four sites. A detailed description of the CERES–maize model of DSSAT is available in Jones and Kiniry (1986). However, briefly, the model utilizes data on daily weather (rainfall, minimum and maximum temperature, solar radiation), soil profile data, crop management and crop genetic coefficients to simulate development, growth and yield of maize as well as soil processes. Plant development is determined by thermal time while growth is a function of solar radiation, water and nutrient stresses and sub-optimal temperature. The soil organic module simulates decomposition processes that determine nitrogen availability for plant uptake. Further details are given in Porter et al. (2010). Additional sources of nitrogen are from fertilizer applications. The nutrient subroutine is closely linked to the soil water sub-routine which describes soil water availability, as well as movement of nutrients within the soil. Soil water dynamics is simulated by describing water flow across layers, plant water uptake, soil evaporation, drainage, with precipitation and or irrigation as water input. Soil water content varies between the lower limit (LL) and the saturated water limit (SAT). Excess water beyond the drained upper limit (DUL) drains to the next soil layer below. Soil surface run-off in the water sub-routine is simulated using a modified USDA-Soil and Conservation Service (SCS) curve number method (Williams, 1990). Priestly-Taylor/ Ritchie (1998) approach was used to simulate evapotranspiration.

### Maize model calibration and evaluation

Apart from the weather data, other inputs required for modeling in DSSAT are crop management, soil, and cultivar specific parameters (genetic coefficients). In Ghana, CERES-maize model has been used to assess the effects of agronomic practices such as fertilizer and manure application on maize yields at some locations including northern Ghana (Dzotsi et al., 2010; Fosu et al., 2012; Fosu-Mensah et al., 2012; MacCarthy et al., 2012). The current version of the model used in this study was derived from a re-calibration for *Obatanpa*, a medium (105–110 days) maturity duration variety commonly grown throughout Ghana including the northern regions. The calibration procedure began with phenology, followed by growth parameters and yield parameters. Data from maize experiments carried out under fully irrigated conditions at the Soil and Irrigation Research Centre (SIREC) of the University of Ghana (located at Latitude 7°N) in 2014 for three planting dates was used to re-calibrate the DSSAT maize model. Though the northern regions of Ghana are located in the 9–11°N bracket, the SIREC calibration results were considered valid because the experiments were conducted under non-limiting water and nutrient conditions, and more so, temperature variations across Ghana are normally small. The calibration data were from experiments in which planting density was 6.25 plants per m^2^, nitrogen application was 120 kg N ha^−1^, split applied at 10 and 36 Days After Emergence (DAE), P applied at 45 kg ha^−1^ at 10 days after emergence and 5 t ha^−1^ of manure was applied.

For the experiment at SIREC, soil profile data were taken from the experiment plots and the weather was recorded by SIREC Weather Station located about 100 m away from the experimental plots. Crop and weather data were used to determine the genetic coefficients for the *Obatanpa* variety such as the thermal time units (P1, P5, and PHINT) for plant development (Table 1). The photoperiod sensitivity (P2) was set to zero. The maximum grain number per cob (G2) was also determined from the experiment.

**Table 1:** **Genetic coefficients for the ***Obatanpa*** maize variety**.

**Genetic co-efficient**	**Definition**	**Value**
P1	Thermal time from seedling emergence to the end of the juvenile phase (expressed in degree days above a base temperature of 8°C) during which the plant is not responsive to changes in photoperiod.	280
P2	Extent to which development (expressed as days) is delayed for each hour increase in photoperiod above the longest photoperiod at which development proceeds at a maximum rate (which is considered to be 12.5 h).	0
P5	Thermal time from silking to physiological maturity (expressed in degree days above a base temperature of 8°C).	750
G2	Maximum possible number of kernels per plant.	591
G3	Kernel filling rate during the linear grain filling stage and under optimum conditions (mg/day).	7.5
PHINT	Phylochron interval; the interval in thermal time (degree days) between successive leaf tip appearances.	45

The performance of the model in adequately representing observed data was assessed using the Root Mean Square Error (RMSE), Willmott's *d*-index and coefficient of determination (*R*^2^). The *RMSE* was defined as:

(1) $$\begin{array}{l} RMSE={\left[\frac{1}{n}\sum {\left(yield_{simulated_{i}}-\;yield_{observed_{i}}\right)}^{2}\right]}^{0.5} \end{array}$$

The lower the *RMSE*, the better the model performance and its minimum value of zero implies a perfect model performance. The Willmott *d*-index (Willmott, 1981) defined as:

(2) $$\begin{array}{l} d-index=1-\frac{\sum_{i=1}^{n}\left(Observed_{i}-Simulated_{i}\right)}{\begin{array}{l} \sum_{i=i}^{n}(\left|Simulated_{i}-Mean_{observed}\right|\\ \;+\left|Observed_{i}-Mean_{observed}\right|) \end{array}} \end{array}$$

The *d*-index ranges from 0 (implies model predictions are similar to observed mean) to 1 (implies a perfect model performance). The calibration showed that anthesis and physiological maturity were well simulated with RMSE of 2.4 and 2 days respectively. Grain yield was simulated with RMSE of 275 kg ha^−1^ and a Wilmott *d*-index of 0.86 and total biomass calibrated with RMSE of 440 kg ha^−1^, Wilmott *d*-index of 0.91.

**Figure 3:**
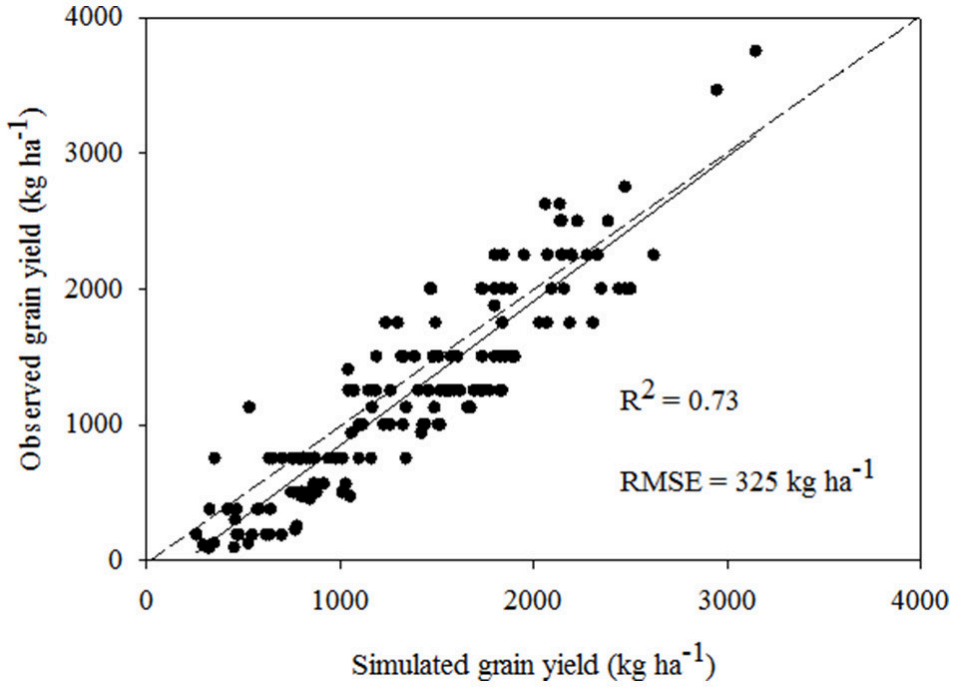
**Comparison of observed and simulated maize grain yield from farmers' fields under different management practices in northern Ghana for the years 2010 and 2014**.

The calibrated model was validated for the northern locations of Ghana (Tamale: Guinea savanna and Navrongo: Sudan savanna) using data from an agronomic survey conducted in 2014 on 185 farms (MacCarthy et al., 2015). The survey documented soil types, maize planting dates, planting density, fertilizer and manure application rates and dates and maize yields. For this dataset, maize yields ranged from about 250 kg ha^−1^ to about 3800 kg ha^−1^, giving a wide spread over varying management conditions. Information collected from farmers were cross-checked with on-station trial data from Savanna Agricultural Research Institute (SARI) at Nyankpala, near Tamale and extraneous data were left out. The survey revealed that the planting window spanned 3 months (May to July) and the average farmer-level fertilizer application rate was 30 kg N ha^−1^. Soil survey data published by the Soil Research Institute of Ghana served to provide some information such as texture and bulk density. Additionally, soils sampled from farmers' fields were used for the determination of other model required properties such as pH and organic carbon content. The weather data for the survey year was obtained from the Ghana Meteorological Agency Stations at Tamale and Navrongo. The simulated yields agreed satisfactorily with the observed (Figure 3) with RMSE of 325 kg ha^−1^, *R*^2^ = 0.73 and Willmott's *d*-index = 0.68. Thus, the maize model was considered reliable and therefore used to simulate crop yield in response to different soil fertility management and varied weather conditions in this study.

### Model application studies at the 4 study locations

#### Soil data

The soil data required for the maize yield simulation for the 4 study locations were obtained from previous soil surveys and sampling campaigns carried out by the authors. The data collected include soil depth, organic carbon, bulk density, and soil texture. Soil water characteristics such as Lower Limit, (LL), DUL, and Saturated Water Content (SAT) were derived from pedo-transfer functions embedded in the DSSAT model shell. The summary of the soil data for the locations is presented in Table 2.

**Table 2:** **Soil Data at the Study Sites used for the simulations in Northern regions in Ghana**.

**Site**	**L cm**	**LL cm^3^/cm^3^**	**DUL cm^3^/cm^3^**	**SAT cm^3^/cm^3^**	**BD g/cm^3^**	**OC %**	**pH**
Tamale	15	0.09	0.18	0.36	1.34	0.41	5.1
	30	0.09	0.18	0.36	1.64	0.38	5.3
	45	0.13	0.19	0.36	1.70	0.24	5.7
	60	0.13	0.19	0.36	1.78	0.10	6.2
	75	0.13	0.23	0.36	1.8	0.03	6.2
Damongo	15	0.092	0.176	0.359	1.43	0.45	5.5
	30	0.092	0.176	0.359	1.45	0.37	5.3
	45	0.113	0.192	0.36	1.45	0.37	5.3
	60	0.114	0.192	0.36	1.45	0.37	5.3
Bawku	15	0.06	0.135	0.387	1.56	0.39	5.1
	30	0.072	0.145	0.38	1.58	0.36	5.3
	50	0.085	0.159	0.388	1.56	0.32	5.3
Wa	22	0.058	0.126	0.46	1.36	0.37	5.7
	40	0.074	0.146	0.431	1.44	0.29	6
	58	0.114	0.196	0.45	1.39	0.22	6.1

*LL, Lower limit; DUL, drained upper limit; SAT, Saturated water content; BD, Bulk density; OC, Organic carbon; L is layer depth. Data source: Soil physical data obtained from Soil research Institute of Ghana. Soil organic carbon and pH were measured in 2014*.

#### Weather data and determination of ENSO-Optimal planting date relationships

Two types of weather data were collected for this study. First, the 30-year (1980–2009) historical daily data (rainfall, minimum and maximum temperature, and solar radiation) were obtained from the Ghana Meteorological Agency for each location. These data were used for maize yield simulations. Second, ENSO data; specifically, the Sea Surface Temperature (SST) anomalies for the NiNO3 region in the tropical Pacific for the same time period (1980–2009) were obtained from the website of the International Research Institute for Climate Prediction, NY, USA. The SST anomalies were sorted into 3 ENSO phases: El Niño, Neutral and La Nina, as defined by the Japan Meteorological Agency. Within the 30 year period, there were 5 El Niño, 9 La Nina and 16 Neutral years. A simple correlation analysis was used to establish relationships between the FMA SST anomalies and seasonal rainfall for each location.

Given that one of the major aims of this study was to employ ENSO for forecasting the optimum planting date, about 40% (13 years) of the 30 years were selected as training data set to derive relationships between the pre-season FMA ENSO and the optimum planting dates. The selected years were 1981, 1984, 1986, 1987, 1991, 1992, 1995, 1996, 1997, 2000, 2003, 2004, and 2008 and this included 3 El Nino, 7 Neutral and 3 La Nina years. The remaining years served as the test data. The selection of the years followed stratified random approach, ensuring that each ENSO phase was included in the training data set. The number of years selected for each ENSO phase also varied with the size of the phase. Based on the planting window information from the farmer survey, maize yields were simulated for each site and for 13 training years from May 15 to July 15 at weekly time intervals under farmer practice of 30 kg N ha^−1^ fertilizer applicate rate. For a given season, planting within the week was effected anytime the soil moisture conditions were adequate. The planting date that resulted in the highest seasonal yield was identified as the “optimal” planting date. For the training dataset, the planting date that produced the highest seasonal for a given year was identified as the “optimal” planting date for that year. Using regression analysis, equations were derived between the FMA SST anomaly and the “optimal” planting dates for each site.

#### Assessing integrated farm management strategies

The ENSO-based derived equations in Section Weather Data and Determination of ENSO-Optimal Planting Date Relationship were used to predict the optimal planting dates for the remaining 17 (test) years for several farm management strategies or scenarios summarized in Table 3. These scenarios were derived based on documentations of the farmer survey. The scenarios included two planting date approaches: (i) ENSO-targeted and (ii) conventional farmer practice; two levels of N application: (i) 30 kg N ha^−1^ (average amount used in a survey) for Farmer Practice (ii) 60 kg N ha^−1^ (recommended rate for farmers in study region) for Enhanced Farmer Practice, without and with manure application of 1000 kg ha^−1^ (which is the average amount applied in the study area).

**Table 3:** **Farm management strategies simulated**.

**Scenario**	**Description**
Low-input farmer practice (LFP)	For this scenario, the planting density was set to 3.5 plants m^−2^ which is commonly observed on farmers' fields and the nitrogen application was 30 kg N ha^−1^. Maize yields were simulated starting from the 15th of May to 15th July at weekly intervals for each of the 4 sites. Planting was effected in each week when soil moisture in the top 30 cm of the soil attained between 60 and 100% moisture conditions.
Enhanced low-input farmer practice (ELFP)	This scenario is similar to 1, except that farmers receive seasonal weather forecast to choose the optimum planting date using the ENSO-based equations derived above.
Medium-input farmer practice (MFP)	For this scenario, the planting density was increased to 6.6 plants m^−2^ which is the recommended plant density for the sites with 60 kg ha^−1^ N (optimum N requirement). As for 1, maize yields were simulated starting from the 15th of May to 15th July at weekly intervals for each of the 4 sites.
Enhanced medium-input farmer practice (EMFP)	This scenario is similar to scenario 3 except that the planting date was ENSO-based (as for 2).
Improved low-input framer practice (ILFP)	Scenario 1 + 1000 kg ha^−1^ manure.
Improved enhanced low-input framer practice (IELFP)	Scenario 2 + 1000 kg ha^−1^ manure.
Improved medium input farmer practice (IMFP)	Scenario 3 + 1000 kg ha^−1^ manure.
Improved enhanced medium input farmer practice (IEMFP)	Scenario 4 + 1000 kg ha^−1^ manure.

**Figure 4:**
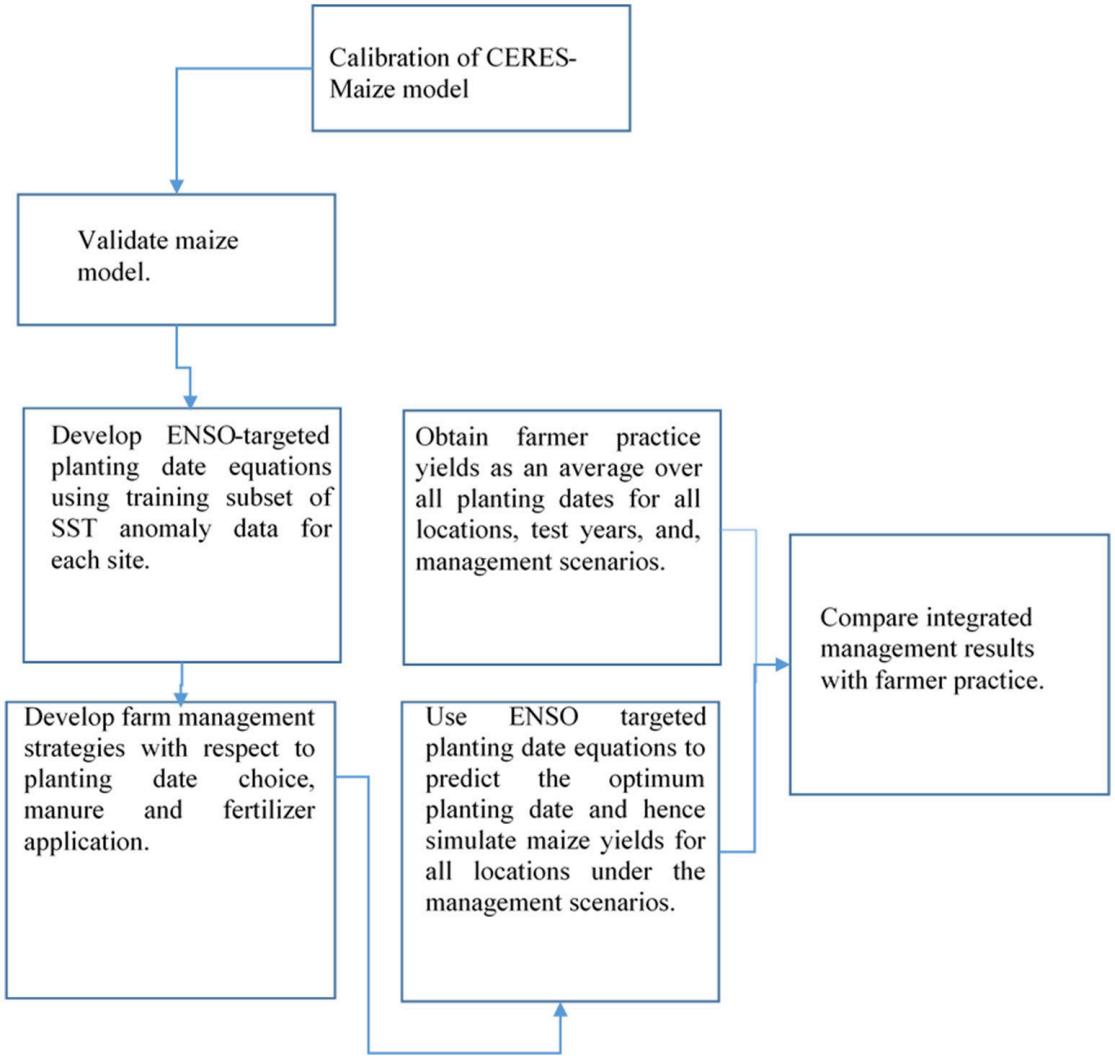
**Flowchart illustrating the chronology of activities undertaken in this study**.

For a given scenario, the simulated yield corresponding the ENSO-based predicted optimal planting date was compared with that of the conventional planting date by the farmer. The latter was derived as an average of yields over all planting dates, because different farmers plant at different times throughout the whole planting window, and there was no information to determine their preferred planting. Moreover, farmers are unlikely to adhere to any particular planting date in all years. A flowchart showing the sequence of determinations is shown in Figure 4.

Comparisons between the different scenarios were based on summary statistics of maize yield under various scenarios and Student *t*-test with significance level set at 5%. A simple ratio of grain yield to the simulated cumulative evapotranspiration (ET) was used an index of water productivity (or Water Use Efficiency: WUE). The partial factor productivity of nitrogen (or the Nitrogen Use Efficiency: NUE) was also defined as the ratio of grain yield to N applied. For each location the WUE and NUE for the various management scenarios were expressed in terms of Cumulative Distribution Function (CDF) (Anderson et al., 1977).

## Results

### ENSO-Rainfall and optimal planting date relationships

The correlation coefficient between the FMA SST and the seasonal rainfall were −0.43 (*p* = 0.02) for Tamale, −0.44 (*p* = 0.01) for Damongo, −0.35 (*p* = 0.08) for Wa, and −0.20 (*p* = 0.25) for Bawku. The negative relationship indicated that rainfall increased with the negative ENSO phase (La Nina) as depicted in Figure 1. The correlations were weak at Wa and Bawku.

**Figure 5:**
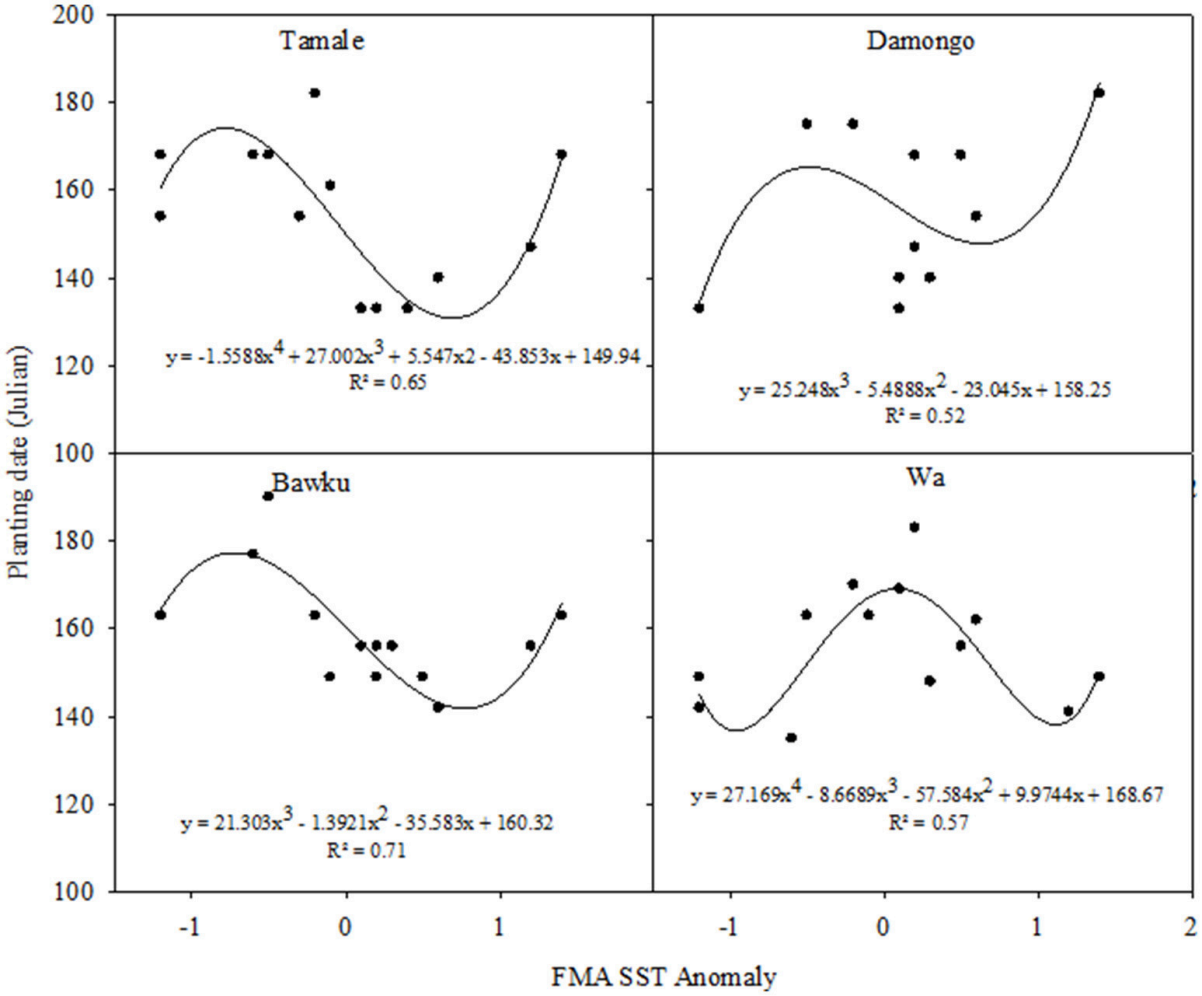
**Relationships between the FMA SST anomaly and optimum planting date (Julian) for the study sites; Tamale, Damongo, Bawku, and Wa**. FMA and SST are February March April and Surface Sea Temperature respectively.

The relationships between the FMA SST anomalies and optimal planting dates were non-linear and followed polynomial functions for all the sites (Figure 5). The relationships for Bawku and Damongo could be described by cubic polynomials whereas those of Wa and Tamale fitted polynomials of the 4th degree. There were no consistent patterns for all locations, even though, early optimum planning date appeared to be associated with negative SST anomalies (La Nina). At Tamale, optimum planting dates for La Nina were from julian Days 150 to 170 (late May to mid June), and similar optimal planting date could be made for El Niño. The optimum planting window for the Neutral ENSO phase had a wide range (Julian Day 130–180; late May to late June). The curve fitted the data quite well with an *R*^2^ = 0.65. At Damongo, not only was the curve fit somewhat poor (*R*^2^ = 0.52) but there was also no clear pattern. The relationship between ENSO and optimum planting date at Bawku followed that for Tamale and the curve fit quite well (*R*^2^ = 0.71). For Wa, early optimum planting dates were associated with La Nina and El Niño (Julian Days 130 to 150; early May to late May), whereas late optimum planting was associated with the Neutral ENSO phase. The curve fitting was also satisfactory (*R*^2^ = 0.57).

### Evaluation of farm management strategic options

The major goal of this study was to investigate the extent to which the combination of ENSO-targeted planting date choice, soil organic resource management and fertilizer application could improve and sustain maize production in the northern regions of Ghana. Our results showed that except for Tamale, the ENSO-targeted planting date strategy resulted in significant maize yields (Table 4). At all locations except Tamale, mean simulated yields based on the ENSO-based optimum planting dates were higher than farmer planting under both 30 and 60 kg N ha^−1^ application rates. Yield increases were between 6 and 19% and between 6 and 23%, for the low and high fertilizer application rates, respectively. Generally, the number of times (Hits) the ENSO-based planting date choice resulted in higher yields than the farmer planting date were as high as 80% for the Bawku site but somewhat lower at Tamale and Damongo (65%).

**Table 4:** **Simulated maize yields at the various locations for ENSO-targeted planting and farmer chosen planting dates at two levels of nitrogen fertilization in four sites in northern Ghana**.

**LOCATION**								
**Statistics**	**Bawku**		**Wa**		**Damongo**		**Tamale**	
	**Farmer**	**ENSO**	**Farmer**	**ENSO**	**Farmer**	**ENSO**	**Farmer**	**ENSO**
**A. 30 kg N ha^−1^**								
Mean (kg ha)	854	1014	1087	1212	1046	1111	1305	1384
Maximum (kg ha^−1^)	1102	1377	1406	1681	1238	1353	1532	1736
Minimum (kg ha^−1^)	653	728	828	894	830	836	1054	895
SD (kg ha^−1^)	139.5	208.6	133.1	205.7	100.6	157	134.7	225.1
CV (%)	15.6	20.5	12.2	17	9.6	14.1	10.3	16.3
*T*-test *p*	*p* = 0.02		*p* = 0.04		*p* = 0.16		*p* = 0.22	
Hit (%)		80		76		65		65
**B. 60 kg N ha^−1^**								
Mean (kg ha^−1^)	1210	1499	1756	1964	1746	1954	2100	2236
Maximum (kg ha^−1^)	1543	1947	2284	2563	2045	2667	2448	3170
Minimum (kg ha^−1^)	876	505	1403	1318	1363	1336	1598	1479
SD (kg ha^−1^)	200.5	354	234	357.7	157.1	371.4	250.5	444.7
CV (%)	16.6	23.6	13.3	18.2	9	19	11.9	19.9
*T*-test *p*	*p* = 0.01		*p* = 0.05		*p* = 0.04		*p* = 0.28	
Hit (%)		80		65		71		71

*CV and SD are coefficient of variation and Standard deviation respectively*.

**Figure 6:**
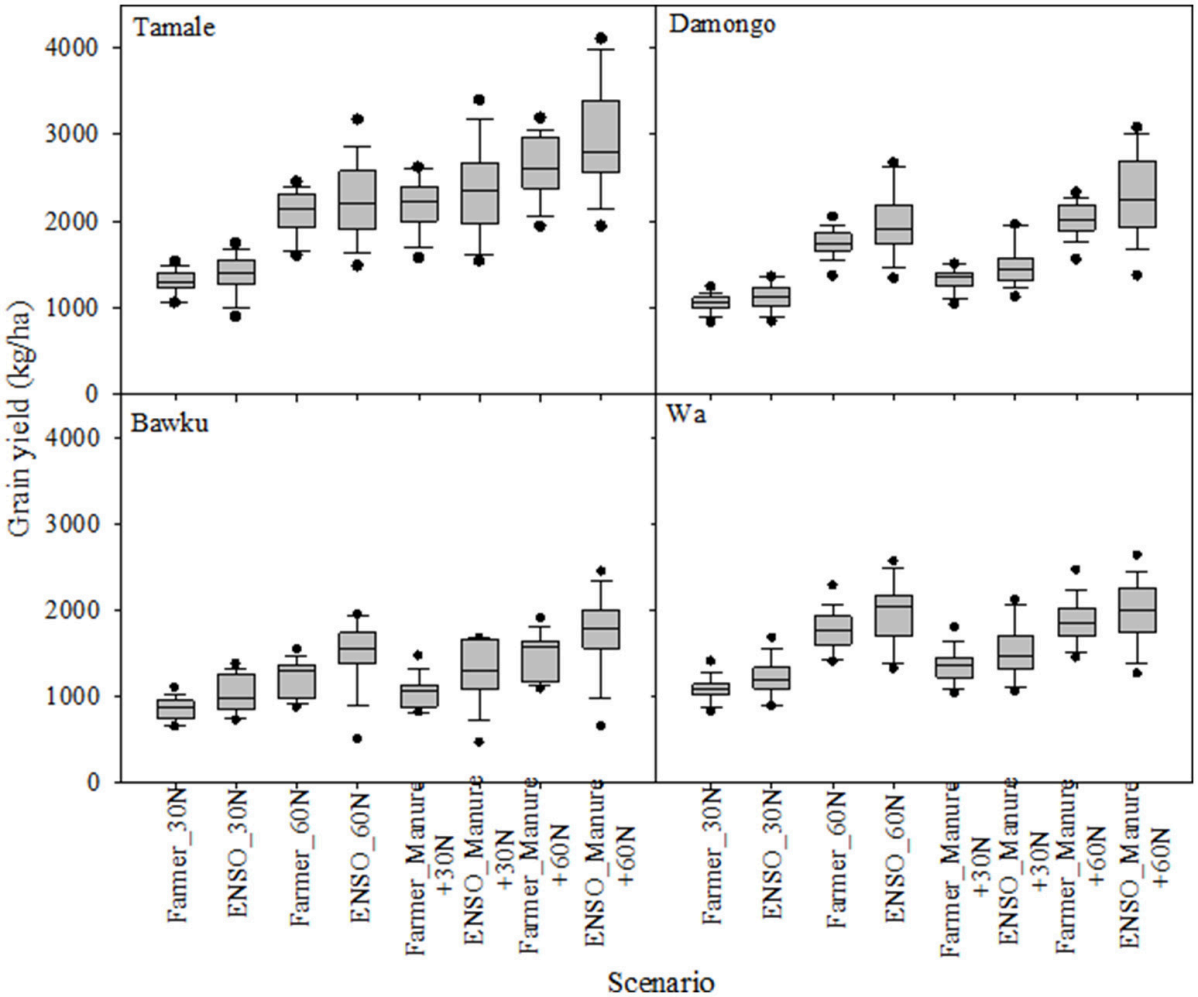
**Comparison of ENSO informed planting and conventional planting at different fertility management strategies at four sites in Northern Ghana**. Each box in the graph shows the distribution of grain yield over the simulation period. The boundary of the box closest to zero indicates the 25th percentile, a line within the box marks the median, and the upper boundary of the box indicates the 75th percentile. Whiskers above and below the box indicate the 95th and 5th percentiles.

The addition of manure further increased the maize yields both under farmer practice and ENSO-based practice, indicating that the combination strategy was still superior (Figure 6). For example, under the farmer practice (30 kg N ha^−1^) at Bawku, addition of manure increased the yield from 854 to 1053 kg ha^−1^ (23%) whereas that for ENSO-targeted planting increased yield by 25% from 1014 to 1273 kg ha^−1^. For the Enhanced farmer practice where 60 kg N ha^−1^ was applied, manure addition under farmer planting date increased the yield from 1210 to 1474 kg ha^−1^ (22%) while ENSO-based planting date increased the yield from 1499 to 1763 kg ha^−1^ (15%). The differences between farmer planting date and ENSO-based planting date were significant (*p* = 0.04). Similar trends could be observed at all locations (Figure 6).

### Water and nitrogen productivity under the different scenarios

**Figure 7:**
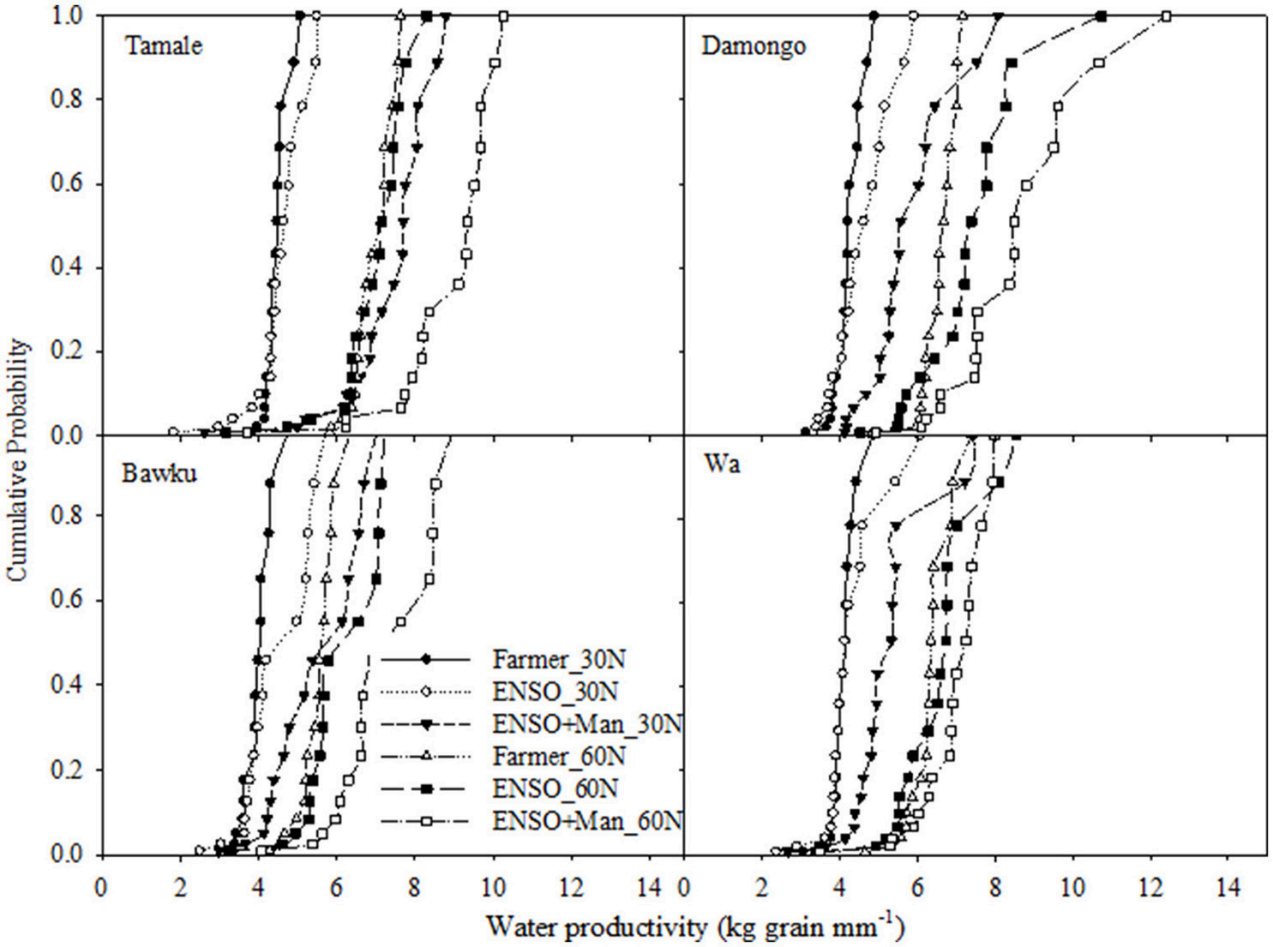
**Frequency distribution of water use efficiency (productivity) factor under ENSO informed and conventional planting strategies with different soil fertility management strategy for the study sites; Tamale, Damongo, Bawku, and Wa**.

The cumulative frequency distribution (CDF) of the WUE are shown in Figure 7 for the various strategies. Strategies whose CDFs fall to the right are considered dominant or preferred. However, where there is incomplete dominance, i.e., where the curves cross, the median maize yield was used to judge strategy performance. Figure 7 shows that at all sites, the lowest water productivity factor was observed for the conventional farmer planting date + 30 kg N ha^−1^ and the highest was for ENSO-targeted planting date, 60 kg N ha^−1^ + manure application of 1000 kg ha^−1^. At Tamale, the WUE ranged from 3.0 to 10 kg mm^−1^, and that for Damongo was from 4 to 9.0 kg mm^−1^. Bawku and Wa had somewhat slightly lower values of 3.5–8.0 kg mm^−1^. Though ENSO-targeted planting date, high N and manure application generally increased the WUE, the variability of the factor also increased considerably under this strategy.

**Figure 8:**
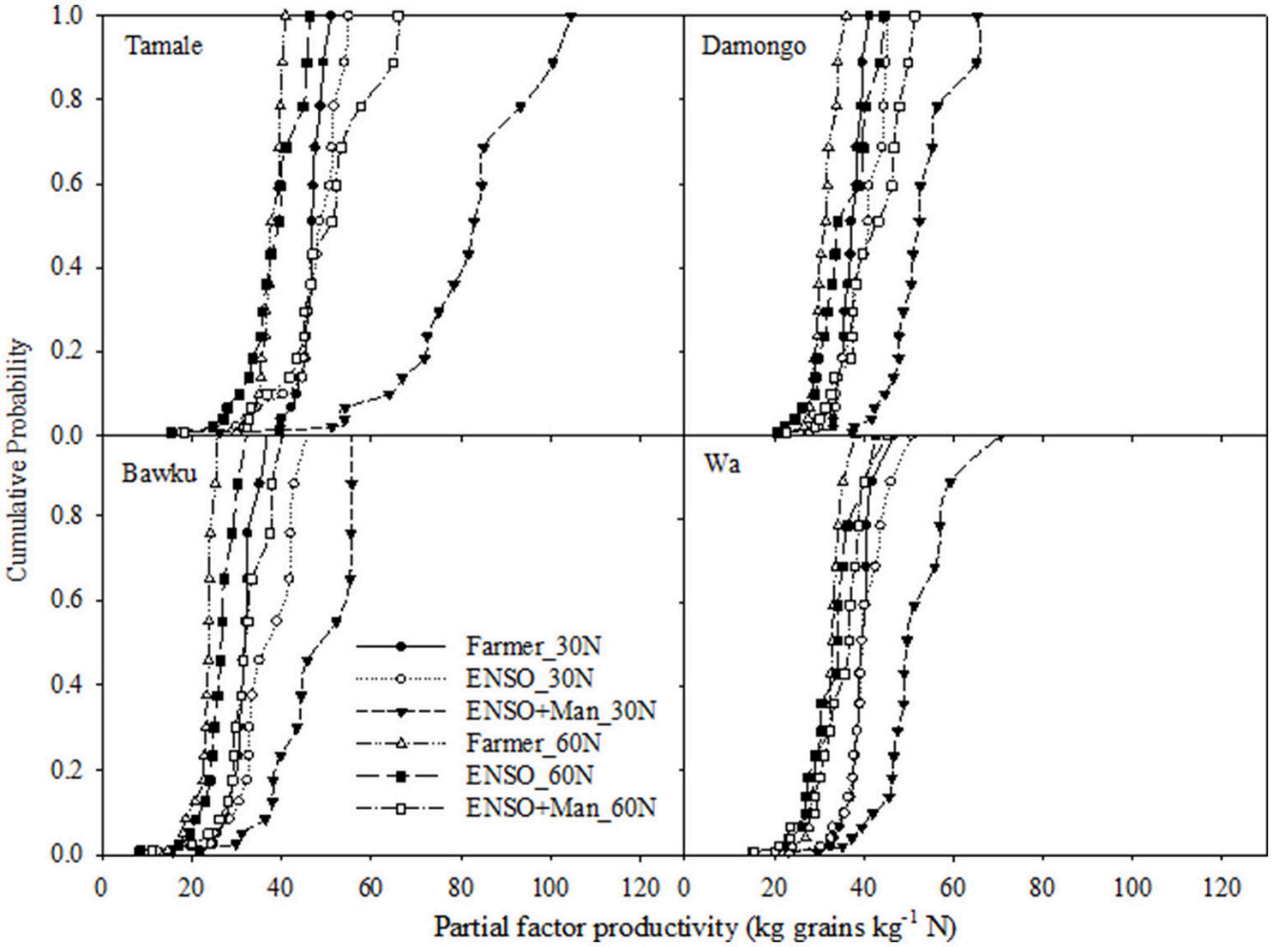
**Frequency distribution of nitrogen use efficiency (productivity) factor under ENSO informed and conventional planting strategies with different soil fertility management strategy for the study sites; Tamale, Damongo, Bawku, and Wa**.

Unlike the water productivity, the highest NUE was generally associated with the ENSO-targeted planting date, 30 kg N ha^−1^ and manure application across sites (Figure 8). Also, the performance of the various strategies were similar at Damongo as the CDFs were close with the median NUE ranging from 25 to 40 kg grain kg^−1^ N. The widest separation of the strategy CDFs was observed at Tamale with the median values ranging from 30 kg grain kg^−1^ N (farmer planting + 30 kg N ha^−1^) to 100 kg grain kg^−1^ N (for ENSO-targeted planting date + 30 kg N ha^−1^ + manure 1000 kg ha^−1^). The magnitude of the productivities, however, varied across sites with the highest median yield obtained for Tamale and the least for Bawku.

## Discussion

There is a need for improving ENSO-based approaches for forecasting seasonal rainfall at the study locations. The relatively low correlation coefficients (−0.2 to −0.43) between seasonal rainfall amounts and the ENSO SST anomalies and the fact that the correlation was only significant for two locations indicates that further research is needed to improve the predictive skill. Furthermore, the literature indicates that for crop growth, not only the seasonal rainfall but also parameters such as onset, and within-season variability of rainfall, among others, are important aspects (Shin et al., 2009; Kassie et al., 2014). Phillips et al. (1998) in their study in Zimbabwe also reported that forecast based on ENSO alone is unlikely to provide adequate information for maize decision making. However, attempts to forecast the season onset from ENSO was not successful in East Africa but it is generally recognized that seasonal climate forecast provides the potential to help farmers decision making (Amissah-Arthur, 2005).

Given the difficulty in adequately predicting the season onset with ENSO, the current study, explored the use of ENSO and DSSAT as decision support tools to select optimum planting date (not onset) and combined these with soil fertility management for sustainable maize production. This was successful in 3 out of the 4 study sites. But, here too, there is need for further research. Though the ENSO selected optimal planting dates resulted in higher yields that farmer planting dates, there was higher yield variability associated with the former. This is undesirable, because yield fluctuations would handicap adoption of new agro-technologies (Hansen, 2005). In effect, farmers may shy away from utilizing ENSO-based planting date strategy.

The ability to target a planting date based on a prior knowledge of the coming season's potential thus offers an opportunity to minimize the risks of missing fertilizer and manure application benefits. The decision on when to sow can also be determined by socioeconomic situations such as availability of draft power. However, if these are not influencing farmers' decision making, the traditional approach of choice of planting date can be minimized. Our approach to minimizing the yield fluctuations, which may be attributed to within season water availability, was to improve soil management to reduce soil water and nutrient loss. With regard to water, Wallace (2000) indicated that on sandy soils as much as 40–50% of rainfall is lost via runoff, deep drainage and soil evaporation. In particular, where plant populations are low as is typical in many traditional farming systems the soil evaporation component may be high, especially soon after rainfall events. At least, the evaporation component should be offset by surface mulching and manure application (Chikowo et al., 2010). Hudson (1994) also showed that for every 1% increase in soil organic matter, the available water holding capacity increases by 3.7, which would imply that even under prolonged dry spells, strategies that increase manure application would result in less water stress. Lal (2006) indicated that maize yield would increase between 30 and 300 kg ha^−1^ for every 1000 kg ha^−1^ increase in soil carbon in the root zone. The simulated yields from this study are within the ranges reported by other studies (Fosu et al., 2012; Ragasa et al., 2014; Naab et al., 2015). Indeed, our simulations in this study showed that the water use efficiency was enhanced under a combination of ENSO-based planting date choice and manure application.

The lingering question is how to access sufficient quantities of organic resources for use as mulch and manure, given that farmers have other uses for crop residues such as fodder and building materials, or in many cases, simply burn off crop residues at the onset of the season. In the case of manure, the challenge with gathering enough manure has to do with the fact that cattle are usually not kept in kraal except in the night, hence significant amount of the manure is not easily available for collection. Also, manure has multiple uses such as building material restricting its availability for large application to farmlands. Research by Adiku et al. (2009) showed that a maize-pigeon pea rotation could generate sufficient residue *in situ* from the pigeon pea fallow which was applied as mulch in every maize growing season and also used to feed livestock. This rotation could sustain the soil carbon in the medium term. Such management practices may form the basis for policies to promote the planting of fast growing leguminous shrubs for residue generation and soil fertility enhancement. Specific policies may be required to incentivize manure application on farms due to the overall soil productivity enhancement. The combination of manure + modest fertilizer application is therefore a soil management practice that is worth exploring as drought tolerance strategy that is within the reach of a farmer. This study has shown that a given foreknowledge of ENSO is useful for farmer-level practical decision making (a decision making tool) in terms of predicting optimal planting time. The approach may not have worked elsewhere but that need not preclude further testing at other locations since ENSO signal strength varies globally. This study provides a first strong evidence that the approach already works in 3 out of 4 locations in Ghana but further work is required to find further support for this approach which should also replicated in other areas with similar climatic conditions.

In summary, our study clearly demonstrated the advantages of the combined planting date choice and soil management, however, there remain challenges that require further research. First, research into seasonal forecasting is still rudimentary in Ghana. Secondly, access to affordably-priced fertilizer and manure continues to elude the traditional farmer. Third, the utility of crop modeling for decision support is still not widespread in many tropical countries. In this study, it could be demonstrated that the CERES maize model enabled the evaluation of maize performance not only over many years but also across many planting dates as well as at several locations. Though model use in decision making has become common place in many industrialized countries, model use in Africa continues to lag behind. Much more support is required to increase modeling not only for research but also for practical decision making and agricultural planning.

## Conclusions

This study employed simulation modeling to assess the value an integrated ENSO-targeted planting date+ tailored soil management strategy for increasing and sustain maize yields in the northern regions of Ghana. It could be shown that the optimum planting date for a given year was predictable from the February-to-April (FMA) SST anomaly for the locations with *R*^2^ ranging from 0.52 to 0.71. The use of ENSO-targeted planting date management strategy was superior to the traditional farmer practice at 3 out 4 locations. The application of a modest fertilizer (60 kg N ha^−1^) and 1000 kg ha^−1^ manure could sustain maize yields at 2360 kg ha^−1^ across years and sites. This was more than 53% yield increase over the traditional farmer practice. In effect, farmer yield levels could be substantially improved and yield fluctuations minimized. The improvement of seasonal rainfall forecasting, affordable fertilizer pricing policy and increasing the availability of organic manures remain challenges that require further research attention.

## Author contributions

DM, SA, BF, FG, and AK: contributed in conceptualizing and designing this study, data acquisition, analysis, interpretation, drafting of the manuscript and reviewing it critically, approved the version to be published.

## Funding

The authors are grateful to the CRP MAIZE, CIMMYT/CGIAR (A4032.09.34), and SARD-SC projects whose financial support made this research possible.

### Conflict of interest statement

The authors declare that the research was conducted in the absence of any commercial or financial relationships that could be construed as a potential conflict of interest.
